# The global and regional prevalence of hepatitis C and B co-infections among prisoners living with HIV: a systematic review and meta-analysis

**DOI:** 10.1186/s40249-021-00876-7

**Published:** 2021-07-01

**Authors:** Hasan Ahmadi Gharaei, Mohammad Fararouei, Ali Mirzazadeh, Golnaz Sharifnia, Marzieh Rohani-Rasaf, Dariush Bastam, Jamileh Rahimi, Mostafa kouhestani, Shahab Rezaian, Mostafa Dianatinasab

**Affiliations:** 1 Department of Epidemiology, School of Public Health and Nutrition, Shiraz University of Medical Sciences, Shiraz, Iran; 2 Department of Health, Faculty of Public Health, Social Determinants of Health Research Center, Gonabad University of Medical Sciences, Gonabad, Iran; 3 Department of Epidemiology and Biostatistics and Institute for Global Health Sciences, University of California San Francisco, San Francisco, CA USA; 4 Department of Epidemiology, School of Public Health, Shahid Sadoughi University of Medical Sciences, Yazd, Iran; 5 Department of Epidemiology, School of Public Health, Shahroud University of Medical Sciences, Shahroud, Iran; 6 Medical School, Yasuj University of Medical Sciences, Yasuj, Iran; 7 Department of Epidemiology and Biostatistics, School of Public Health, North Khorasan University of Medical Sciences, Bojnurd, Iran; 8 Infectious Diseases Research Center, Kermanshah University of Medical Sciences, Kermanshah, Iran; 9 Department of Complex Genetics and Epidemiology, School of Nutrition and Translational Research in Metabolism, Maastricht University, Universiteitssingel 40 (Room C5.570), 6229 ER Maastricht, the Netherlands

**Keywords:** Hepatitis B, Hepatitis C, HIV, AIDS, Prisons, Prevalence, Co-infection

## Abstract

**Background:**

Hepatitis B virus (HBV) and hepatitis C virus (HCV) infections are common among individuals with human immune deficiency virus (HIV) infection worldwide. In this study, we did a systematic review and meta-analysis of the published literature to estimate the global and regional prevalence of HCV, HBV and HIV coinfections among HIV-positive prisoners.

**Methods:**

We searched PubMed via MEDLINE, Embase, the Cochrane Library, SCOPUS, and Web of science (ISI) to identify studies that reported the prevalence of HBV and HCV among prisoners living with HIV. We used an eight-item checklist for critically appraisal studies of prevalence/incidence of a health problem to assess the quality of publications in the included 48 cross-sectional and 4 cohort studies. We used random-effect models and meta-regression for the meta-analysis of the results of the included studies.

**Results:**

The number of the included studies were 50 for HCV-HIV, and 23 for HBV-HIV co-infections. The pooled prevalence rates of the coinfections were 12% [95% confidence interval (*CI*) 9.0–16.0] for HBV-HIV and 62% (95% *CI* 53.0–71.0) for HCV-HIV. Among HIV-positive prisoners who reported drug injection, the prevalence of HBV increased to 15% (95% *CI* 5.0–23.0), and the HCV prevalence increased to 78% (95% *CI* 51.0–100). The prevalence of HBV-HIV coinfection among prisoners ranged from 3% in the East Mediterranean region to 27% in the American region. Also, the prevalence of HCV-HIV coinfections among prisoners ranged from 6% in Europe to 98% in the East Mediterranean regions.

**Conclusions:**

Our findings suggested that the high prevalence of HBV and HCV co-infection among HIV-positive prisoners, particularly among those with a history of drug injection, varies significantly across the globe. The results of Meta-regression analysis showed a sliding increase in the prevalence of the studied co-infections among prisoners over the past decades, rising a call for better screening and treatment programs targeting this high-risk population. To prevent the above coinfections among prisoners, aimed public health services (e.g. harm reduction via access to clean needles), human rights, equity, and ethics are to be seriously delivered or practiced in prisons.

*Protocol registration number*: CRD42018115707 (in the PROSPERO international).

**Graphic abstract:**

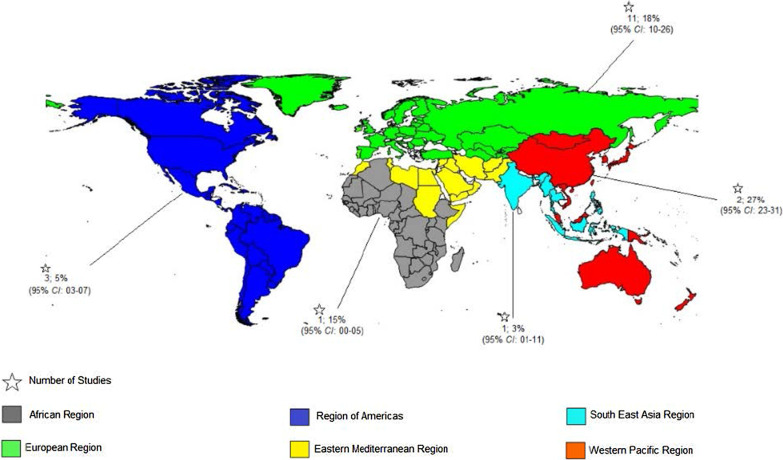

**Supplementary Information:**

The online version contains supplementary material available at 10.1186/s40249-021-00876-7.

## Background

Globally, hepatitis C virus (HCV) and hepatitis B virus (HBV) infections are major health problems with considerable morbidity and mortality due to serious complications [1]. This issue is even more serious among human immunodeficiency virus (HIV) infected individuals [2, 3]. The co-infections may accelerate the disease progression and raise the risk of severe liver diseases or mortality, and also cause complications in the treatment of HIV and hepatitis coinfection [4]. The global prevalence rates of HCV and HBV infections among HIV-positive individuals are estimated to be 2.4% and 7.6% respectively [2, 5].

Among different segments of a population, prisoners are at a higher risk of HIV, HCV, and HBV infections due to risky behaviors such as drug use and high-risk sexual practices [6, 7]. A review of literature from 2005 to 2015 among prisoners showed that the global prevalence of HIV, HCV, and HBV in prisoners are about 3.8%, 15.1%, and 4.8% respectively [6]. The highest prevalence of HIV/HCV co-infection was reported for people who inject drugs (PWID) followed by men who have sex with men (MSM) [8]. The prevalence of HIV was the highest among prisoners in sub-Saharan Africa, in Europe, and central Asia respectively [8, 9]. The highest HBV prevalence among prisoners was reported from West and Central Africa [8]. The reports also suggested a high variation in the prevalence of HIV and viral hepatitis infections among prisoners worldwide.

Prisons provide both challenges and opportunities to HIV and viral hepatitis control programs. If effective control measures are not applied in the prisons, these places can become a place for further spread of the above infections among prisoners, to their family, and a wider community upon their release [9]. The previous outbreaks of HIV, HCV and other infections in prison settings highlight the importance of the above issue [8]. On the other hand, prisons can provide valuable opportunities regarding the diagnosis and treatment of health conditions such as the screening of HIV, HCV, and HBV that would have not otherwise been detected in such high-risk minority populations. An example of successful intervention programs targeting the above infections among prisoners is the “community-based needle and syringe programs” in Australia [10]. Australia’s HIV infection prevalence of almost zero in people who inject drugs in and out of prison can be traced back to the introduction of the programs in 1986, which prevented an estimated 25 000 cases of HIV in people who inject drugs [11].

Despite the fact that the prevalence of blood-borne hepatitis viruses and HIV is expected to be very high in correctional facilities around the world [12], only a few regional studies assessed the prevalence of these infections in these centers [11–13]. Moreover, limited evidence are available on the global status of HCV and HBV coinfection among HIV-positive prisoners. To address this important issue, we did a comprehensive systematic review and meta-analysis of the published literature to estimate the global and regional prevalence of HCV, HBV and HIV coinfections among the above mentioned high-risk population.

## Methods

### Protocol and registration

We designed our systematic review and meta-analysis according to the Meta-analysis of Observational Studies in Epidemiology checklist [14] and PRISMA (preferred reporting items for systematic reviews and meta-analyses) standards [15, 16], under a registered protocol (registration number CRD42018115707) [17].

### Search strategy and selection criteria

We did a comprehensive systematic search of the literature to find cross-sectional and cohort studies that investigated the prevalence and incidence of HCV or HBV in prisoners living with HIV. We searched PubMed/MEDLINE, Embase, the Cochrane Library, SCOPUS, and Web of science (ISI) databases by combining three sets of related MeSH and Non-MeSH terms: (1) “hepatitis B” OR “HBV” OR “hepatitis C” OR “HCV”, (2) “HIV”, “AIDS”, “acquired immune deficiency syndrome” and (3) “prison* OR concentration camps OR incarcerate* OR penitentiar* OR jail to find relevant studies that were published until December 2020, with no limitation regarding language.

Studies were eligible for our review if they met the following criteria (1) original studies with cohort or cross-sectional designs; (2) study that recruited prisoners, and (3) studied that diagnosed HIV, HCV, and HBV infections with standard laboratory tests. Three authors independently reviewed the studies and discrepancies were resolved by discussing with the fourth author. The references of eligible articles were also manually reviewed for other possibly related articles that were not found in the electronic search.

### Quality assessment

The eight-item checklist for critically appraisal studies of prevalence/incidence of health problems [16] was used to examine the quality of eligible studies by two independent investigators (HA and JR). The range of the total score was from 0 (the lowest possible quality) to 8 (the highest possible quality); the details of this tool are provided in the Additional file 1. The quality of the studies is categorized as high (score ≥ 7), medium (score between 4 and 6), or low (score < 4).

### Data extraction

Two of the co-authors independently extracted the following data from included studies; author’s name, study year, study design, country and region, mean age of the participants, the number of prisons, the total number of prisoners who had HIV infection, and the frequency and prevalence of HCV or HBV in those prisoners living with HIV. The extracted data were compared and the reviewers discussed discrepancies to reach a consensus. If the full text of a study was unavailable or if the reported data was missing key information, we contacted the authors by email at least twice, one week apart. In case that we did not hear from the authors, we sent an email to the publishers (e.g. Elsevier or Wiley Online Library) to help.

### Statistical analysis

The number of individuals with HCV or HBV infections in prisoners living with HIV and the total number of prisoners living with HIV were used to calculate the prevalence of coinfection (in logit form) and its corresponding standard error (SE). The summary prevalence with 95% *CI* was obtained using a random-effects model. Cochran’s Q test was used to identify the heterogeneity of the results, and it was quantified using the *I*^2^ statistic. *I*^2^ statistic > 50% or Q statistics with *P* < 0.10 were considered as a significant between-study heterogeneity. Subgroup analysis based on the region of the study was performed to explore possible sources of heterogeneity. A meta-regression analysis was conducted to explore the association between the study year and the difference in HCV or HBV prevalence among prisoners living with HIV. A jack-knife sensitivity analysis was conducted by removing the studies from meta-analyses one by one. We also evaluated the publication bias using Begg's funnel plots and the asymmetry tests (Egger's and Begg's test). We further used free World Shapefiles to design maps (available at: https://tapiquen-sig.jimdofree.com/english-version/free-downloads/world/) using ArcGIS software (ArcGIS 10.2.2. Esri. 2014-02-27). All statistical analyses were performed using STATA software (StataCorp. 2015. Stata Statistical Software: Release 14. College Station, TX: StataCorp LP.). *P*-values below 0.05 were considered statistically significant.

## Results

### Characteristics of the included studies

**Fig. 1:**
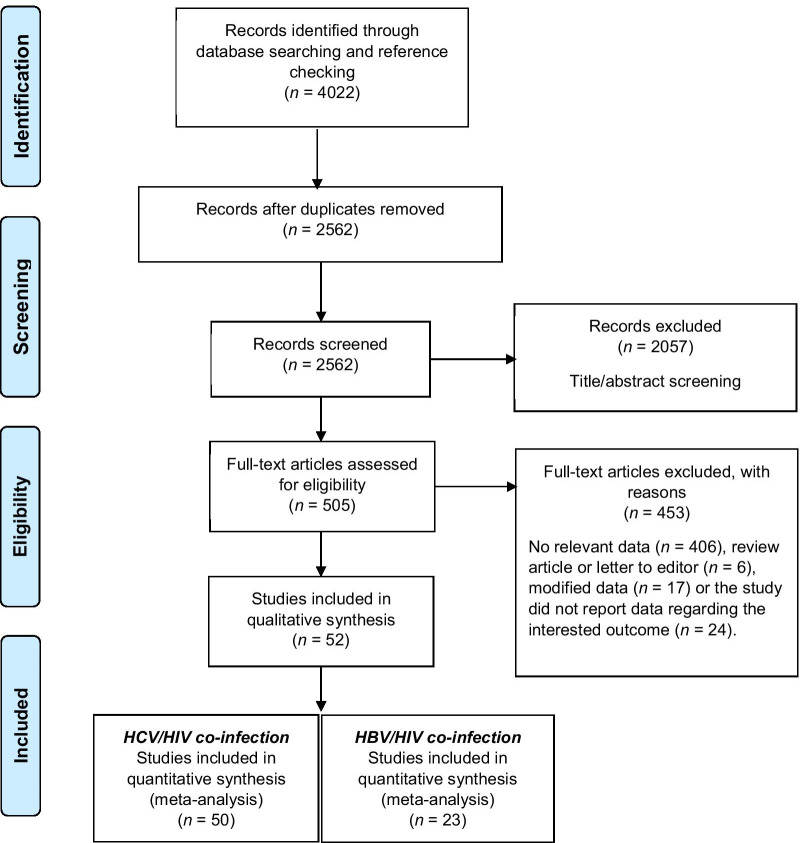
The study flow diagram according to the PRISMA statement

A total of 4022 records were obtained by electronic and manual search (Fig. 1). After removing duplicate records, 2562 records remained for further assessment. We excluded 2057 studies after screening titles and/or abstracts. We read 505 studies and during the full-text review, 453 articles were excluded for different reasons (Fig. 1). In the end, 52 eligible articles were included in our qualitative synthesis and the quantitative meta-analysis [6, 7, 9, 19–31, 33, 35–59, 61–66]. The characteristics of the studies included in this review are presented in Table 1.

**Table 1:** Included studies of HBV, HCV and HBV/HCV co-infections in prisoners with HIV

Row	Author year	Country	WHO regions	Design	Mean age	Number of prisoners living with HIV	Number of people with HBV co-infection	HBV co-infection prevalence (95% *CI*)*	Number of people with HCV co-infection	HCV co-infection prevalence (95% *CI*)*	Quality of study
1	Smith et al. [66] 1991	USA	America	Cross-sectional	29.4	90	3	3.3 (1.0 -9.2)	-	-	High
2	Vlahov et al. [31] 1993	USA	America	Cross-sectional	-	23	-	-	20	86.9 (68.1–95.0)	Medium
3	Bulter et al. [19] 1997	Australia	Western Pacific	Cross-sectional	30.6 (10.1)	408	125	30.6 (26.3 -35.1)	150	36.7 (32.0–42.4)	High
4	Pallas et al. [2, 21] 1999	Spain	Europe	Cross-sectional	27.2 (5.4)	156	5	3.2 (1.0–7.1)	9	5.7 (3.2–10.9)	Medium
5	Massed et al. [20] 1999	Brazil	America	Cross-sectional	30.8	211	-	-	75	35.5 (29.3–41.8)	Medium
6	Guimarães et al. [22] 2001	USA	America	Cross-sectional	30.2	105	-	-	54	51.4 (423–61.4)	High
7	Quaglio et al. [24] 2003	Italy	Europe	Cross-sectional	30 (7)	133	-	-	128	96.2 (91.9–98.0)	Medium
8	Baillargeon et al. [23] 2003	USA	America	Cross-sectional	-	46	-	-	24	52.1 (38.0–65.7)	Medium
9	Solomon et al. [26] 2004	USA	America	Cross-sectional	-	220	-	-	144	65.4 (59.2–71.1)	Low
10	Rui Passadouro et al. [25] 2004	Portugal	Europe	Cross-sectional	32.3	47	38	87.2 (75.0–94.3)	41	80.8 (67.2–90.0)	Low
11	Hoya et al. [27] 2005	Spain	Europe	Cross-sectional	34.2 (6.2)	146	-	-	137	93.8 (88.8–97.2)	Low
12	Babudieri et al. [65] 2005	Italy	Europe	Cross-sectional	36	73	54	74.0 (63.1–83.4)	59	81.2 (70.1–88.0)	Medium
13	Khodabakhsi et al. [28] 2007	Iran	Eastern Mediterranean	Cross-sectional	-	7	1	14.2 (3.0–51.3)	3	42.8 (16.1–75.0)	High
14	Calzavara et al. [29] 2007	Canada	America	Cross-sectional	-	25	-	-	17	68.0 (48.1–83.4)	Medium
15	Poulin et al. [30] 2007	Canada	America	Cross-sectional	-	54	-	-	35	64.8 (51.3–76.1)	Medium
16	Baillargeon et al. [32] 2008	USA	America	Prospective cohort	-	6168	312	5.0 (4.9–6.3)	2275	36.8 (36.0–38.1)	Low
17	Barros et al. [33] 2008	Portugal	Europe	Cross-sectional	-	41	-	-	20	48.7 (34.0–59.7)	High
18	Pontali et al. [1, 34] 2008	Italy	Europe	Prospective cohort	-	164	11	6.7 (4.3–12.1)	149	90.0 (85.3–94.2)	Low
19	Davoodian et al. [36] 2009	Iran	Eastern Mediterranean	Cross-sectional	35.4 (8.4)	38	3	7.8 (3.0–20.8)	36	94.7 (83.2–99.0)	Low
20	Hennessey et al. [37] 2009	USA	America	Cross-sectional	-	168	14	8.3 (5.2–14.0)	64	38.0 (31.1–46.2)	High
21	Rosen et al. [38] 2009	USA	America	Cross-sectional	-	718	-	-	487	67.8 (63.8 -71.0)	Medium
22	Adoga et al. [64] 2009	Nigeria	Africa	Cross-sectional	29.2	54	8	14.8 (8.0–27.2)	-	-	Medium
23	Chu et al. [35] 2009	Taiwan	Western Pacific	Cross-sectional	31.4 (7.2)	52	6	11.5 (5.0–23.3)	51	98.0 (90.1–99.9)	Medium
24	Sánchez et al. [39] 2009	Taiwan	Europe	Cross-sectional	-	188	-	-	167	88.8 (83.7–93.7)	High
25	Nelwan et al. [41] 2010	Indonesia	South East Asia	Cross-sectional	28.9	63	2	3.1 (1.0–11.2)	52	82.5 (71.3–90.4)	Medium
26	Hosseini et al. [40] 2010	Iran	Eastern Mediterranean	Cross-sectional	-	102	-	-	100	98.0 (93.0–99.4)	Medium
27	Mir-Nasseri et al. [43] 2011	Iran	Eastern Mediterranean	Cross-sectional	35.85	63	3	4.7 (2.0–12.9)	56	88.8 (79.3–95.0)	High
28	Nafees et al. [44] 2011	Pakistan	Eastern Mediterranean	Cross-sectional	-	99	1	1.0 (0.1–6.2)	73	74.7 (64.2–81.0)	High
29	Da Siva et al. [42] 2011	Portugal	Europe	Cross-sectional	34.1 (10.8)	10	-	-	8	80.0 (49.1–94.1)	Low
30	Marco et al. [45] 2012	Spain	Europe	Cross-sectional	35.7 (10.3)	40	2	5.0 (1.0–17.2)	34	85.0 (71.0–93.0)	Medium
31	Prasetyo et al. [48] 2013	Indonesia	South East Asia	Cross-sectional	32.3	18	-	-	15	83.3 (61.0–93.8)	Low
32	Semaille et al. [49] 2013	France	Europe	Cross-sectional	34 (0.56)	26	-	-	2	7.6 (2.3–24.1)	Medium
33	Mourino et al. [46] 2013	Spain	Europe	Cross-sectional	34.4 (7.4)	47	3	6.3 (1.9–17.0)	30	63.8 (50.0–76.4)	High
34	Brandolini et al. [47] 2013	Italy	Europe	Cross-sectional	43.2 (11.18)	67	-	-	60	89.5 (80.2–95.0)	High
35	Azbel et al. [9] 2013	Ukraine	Europe	Cross-sectional	31.9	78	-	-	72	92.3 (84.0–96.5)	High
36	Alvareza et al. [6] 2014	USA	America	Cross-sectional	37.4 (10.9)	119	-	-	47	39.4 (31.0–48.0)	Medium
37	Marco et al.[10] 2014	Spain	Europe	Prospective cohort	39.7 (11)	46	-	-	19	41.3 (28.5–56.0)	High
38	Rodriguez et al. [50] 2014	USA	America	Cross-sectional	39	37	-	-	31	83.7 (69.4–92.3)	Medium
39	Escudero et al. [51] 2014	Afghanistan	Eastern Mediterranean	Cross-sectional	-	39	-	-	37	94.8 (82.7–99.0)	Low
40	Chan et al. [52] 2015	United Kingdom	Europe	Cross-sectional	-	151	5	3.3 (1.0–8.0)	33	21.8 (16.5–29.2)	Low
41	Farhoudi et al. [54] 2016	Iran	Eastern Mediterranean	Cross-sectional	-	85	3	3.5 (1.5–10.2)	50	58.8 (48.0–69.2)	High
42	Sanarico et al. [56] 2016	Italy	Europe	Cross-sectional	-	69	8	11.5.00 (5.6–21.1)	8	11.5 (6.0–20.9)	Medium
43	Pontali et al. [2, 56] 2016	Italy	Europe	Cross-sectional	-	365	25	6.8 (5.1–10.3)	291	89.7 (74.9–84.3)	High
44	Gabriel et al. [53] 2016	Malaysia	Western Pacific	Cross-sectional	35.2 (10.1)	221	-	-	200	90.4 (86.0–94.2)	High
45	Pontali et al. [3, 58] 2017	Italy	Europe	Cross-sectional	45.7	85	2	2.3 (1.3–8.5)	46	54.1 (44.1–64.0)	High
46	Puga et al. [61] 2017	Brazil	America	Cross-sectional	32 (10)	54	-	-	5	9.2 (4.0–20.4)	Medium
47	Retana et al. [62] 2017	USA	America	Cross-sectional	33.28	18	-	-	2	11.1 (3.4–33.1)	Medium
48	Flor et al. [59] 2017	USA	America	Cross-sectional	-	30	-	-	6	20.0 (10.0–37.0)	Medium
49	Baillargeon et al. [7] 2017	USA	America	Cross-sectional	37 (11.3)	2960	-	-	708	23.9 (21.7–25.5)	Medium
50	Akiyama et al. [57] 2017	USA	America	Cross-sectional	-	1197	-	-	331	27.6 (24.8–30.3)	Medium
51	Prestileo et al. [60] 2017	Italy	Europe	Prospective cohort	35.5	30	4	13.3 (5.0–30.2)	16	53.3 (36.5–70.1)	Medium
52	Kivimets et al. [63] 2018	Estonia	Europe	Cross-sectional	34.1 (10.92)	469	-	-	379	81.8 (77.0–84.3)	Medium

*Prevalence was provided per 100 people

Of the 52 included studies, 48 were cross-sectional [6, 7, 9, 19–31, 33, 35–59, 61–66] and 4 were prospective cohort studies [10, 32, 34, 60]. Of all, 50 studies reported HCV infection [6, 7, 9, 10, 19–63, 65] and 23 studies reported either solely HBV infection or both HBV and HCV infections [19, 21, 25, 28, 32, 34–37, 41, 43–46, 52, 54–56, 58, 60, 64–66]. Regarding the study location, 21 studies were conducted in Europe [9, 10, 21, 24, 25, 27, 33, 34, 39, 42, 45–47, 49, 52, 55, 56, 58, 60, 63, 65], 18 were conducted in America [6, 7, 20, 22, 23, 26, 29–32, 37, 38, 50, 57, 59, 61, 62, 66], 2 in South-East Asia [41, 48], 7 in East Mediterranean [28, 36, 40, 43, 44, 51, 54], 3 in Western Pacific [19, 35, 53], and 1 in Africa region [64].

### Assessment of the studies’ quality

Out of the 52 included studies [6, 7, 9, 10, 19–66], we found 17 studies [9, 10, 19, 22, 28, 33, 37, 39, 43, 44, 46, 47, 53, 54, 56, 58, 66] with high quality, 25 studies with medium quality [6, 7, 20, 21, 23, 24, 29–31, 35, 38, 40, 41, 45, 49, 50, 56, 57, 59–65], and 10 studies with low quality [25–27, 32, 34, 36, 42, 48, 51, 52]

### HBV infection

Of the 8449 prisoners with HIV recruited in the selected studies, 628 (7.4%) had HBV infection (Table 1). The highest prevalence of HBV infection among HIV positive prisoners was 81% (95% *CI* 67.0–90.0), which was reported by Rui Passadouro et al. (2004) in Portugal [25] and the lowest prevalence (1%, 95% *CI* 0.0–6.0) was reported by Nafees (2011) in Pakistan [44].

**Fig. 2:**
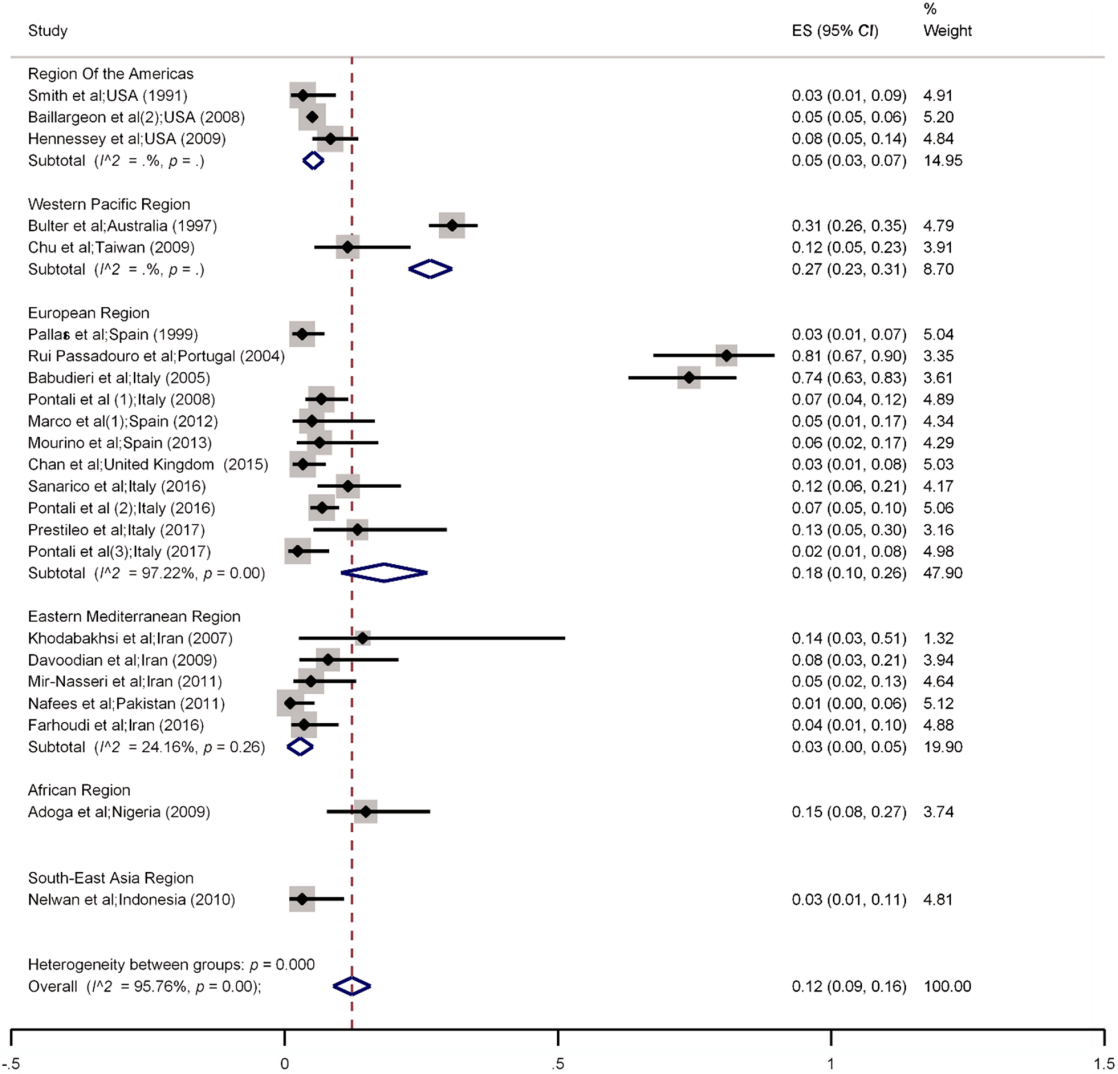
Random-effects meta-analysis of Hepatitis B co-infection among prisoners living with by WHO regions. *ES* Effect size

The overall pooled prevalence of HBV among HIV positive prisoners was 12% (95% *CI* 9.0–16.0, *I*^2^ = 93.8% (*P* < 0.001). Also, twenty three studies [19, 21, 25, 28, 32, 34–37, 41, 43–46, 52, 54–56, 58, 60, 64–66] reported a 15% prevalence of HBV among HIV-positive prisoners with a history of drug injection (pooled prevalence = 12%, 95% *CI* 5.0–23.0), with significant heterogeneity in the results *I*^2^ = 84.8% (*P* < 0.001). Subgroup analysis by WHO regions is provided in Fig. 2 and Additional file 1: Fig. 1. The regional pooled prevalence rates of HBV among HIV-positive prisoners were 27% in the Western Pacific, 18% in Europe, 15% in African regions, 5% in America, 3% in Eastern Mediterranean, and 3% in Southeast Asia respectively.

The results of sensitivity analysis showed no significant difference between all included studies and studies with low-quality scores. The Egger test (*t* = 2.59, *P*-value = 0.01) and funnel plot (Additional file 1: Figs. 2 and 3) indicated a publication bias for studies that reported HBV infection. Also, the results of meta-regression for the prevalence of HBV infection among prisoners living with HIV by the year of publication are provided in Additional file 1: Fig. 4.

### HCV infection

Of the 15 721 recruited HIV positive prisoners, 6858 (43%) had HCV infection (Table 1). The highest and the lowest prevalence of HCV/HIV coinfection was reported in Iran (pooled prevalence = 98%, 95% *CI* 93.0–99.0) [40], and Spain (pooled prevalence = 6%, 95% *CI* 3.0–11.0) respectively [21].

The overall pooled prevalence of HCV infection from the included studies [6, 7, 9, 10, 19–63, 65] was 62% (95% *CI* 53.0–71.0, *I*^2^ = 95.8%, *P* < 0.001). The prevalence of HCV infection among HIV-positive prisoners with a history of drug injection was 78% (95% *CI* 51.0–100) with a significant heterogeneity *I*^2^ = 99.8% (*P* < 0.001).

**Fig. 3:**
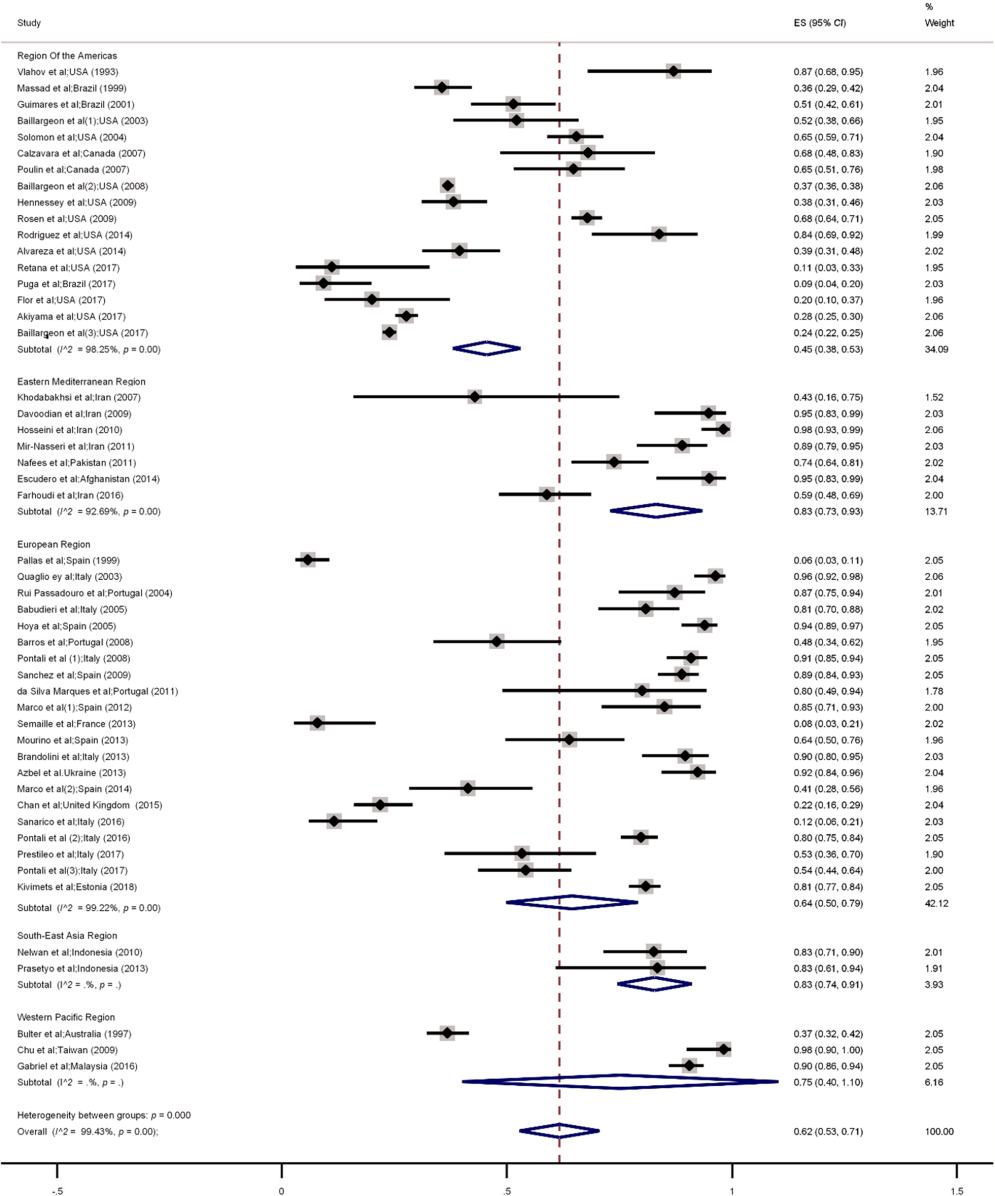
Random-effects meta-analysis of Hepatitis C co-infection among prisoners living with by WHO regions. *ES* Effect size

**Fig. 4:**
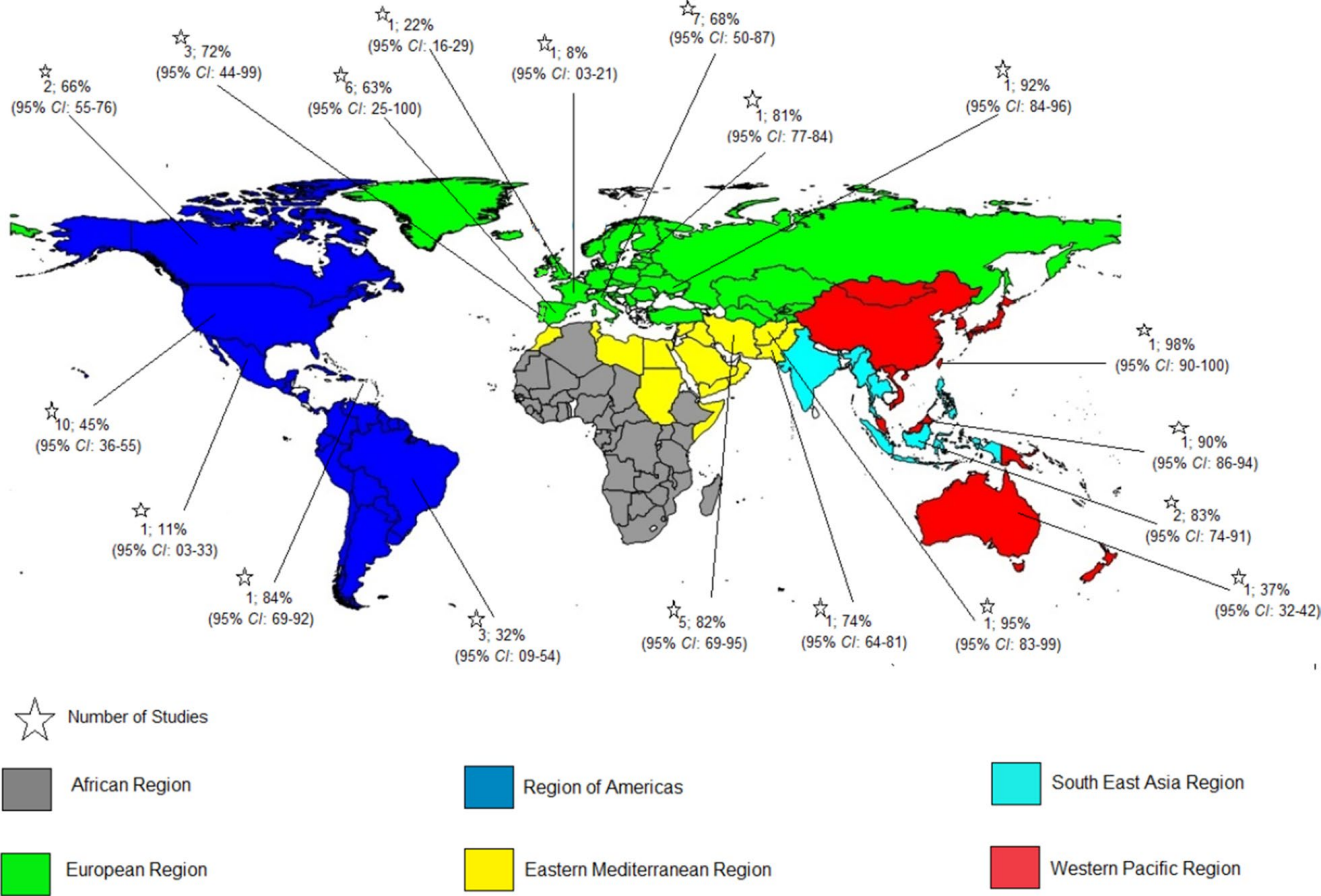
Map of Hepatitis C co-infection among prisoners living with HIV by country within different WHO regions (pooled prevalence and 95% *CI* are shown for each country). Footnote: Prevalence was provided per 100 people. "*" is shown the number of studies.* CI* Confidential interval

The pooled prevalence rates of HCV infection among HIV-positive prisoners were 83% in Eastern Mediterranean, 83% in South East Asia, 75% in Western Pacific, 64% in Europe, and 45% in America respectively (Figs. 3 and 4). No study measured HCV/HIV coinfection among prisoners in the African region.

Meta-regression indicated that heterogeneous results in HCV/HIV coinfection can be significantly attributed to the WHO regions (β = −6.8, *P* = 0.004). No significant association was found for other factors (i.e. study design, study quality, sample size, and years of publication) (Table 2). Results of sensitivity analysis showed no significant difference between all included studies and studies with low-quality scores. Egger test (*t* = 6.6, *P* < 0.001) and funnel plot (Additional file 1: Figs. 5 and 6) indicated a publication bias for studies that reported HCV/HIV coinfection. Meta-regression of the association between the prevalence of HCV/HIV coinfection during 1990–2019 in prisoners and year of study is shown in Additional file 1: Fig. 7.

**Table 2:** Meta-analysis and subgroup analysis of prevalence of HBV, HCV and among prisoners living with HIV

Variable		Number of studies	Pooled prevalence*	95% *CI*	*I*^2^ (%)	Heterogeneity (95% *CI*)	*P* value
*Subgroup analysis for hepatitis B co-infection among prisoners living with HIV*							
WHO regions	Region of the Americas	3	5	3–7	0	1.75 (-3.37 to 6.87)	0.48
	European of region	11	18	10–26	97.22		
	Western Pacific	2	27	23–31	0		
	South-East of Asia	1	3	1–11	0		
	Eastern Mediterranean region	5	3	0–5	24.16		
	African region	1	15	8–27	0		
Study design	Cross-sectional	20	11	6–15	96.30	-6.28 (-33.45 to 20.88)	0.63
	Cohort	3	5	4–7	0		
Quality of study	High	10	8	3–13	94.02	-6.36 (-17.83 to 5.09)	0.26
	Moderate	8	17	5–28	96.24		
	Low	5	18	9–28	97.74		
Sample size	< 50	6	21	0–43	96.64	-10.05 (-30.98 to 10.87)	0.32
	> 50	17	10	7–13	95.34		
Year	< 2005	5	38	16–59	99.04	-8.61 (-15.96 to -1.25)	0.02
	2006–2010	8	6	4–8	34.60		
	2011–2015	4	3	0–6	30.21		
	> 2015	6	5	3–8	54.80		
*Subgroup analysis for Hepatitis C co-infection among prisoners living with HIV*							
WHO regions	Region of the Americas	17	45	38–53	98.25	-6.81 (-11.28 to -2.34)	0.004
	European of region	21	64	50–79	99.22		
	Western Pacific region	3	75	40–100	0		
	South-East of Asia region	2	83	74–91	0		
	Eastern Mediterranean region	7	83	73–93	92.69		
Study design	Cross-sectional	46	62	52–72	99.40	-6.38 (-37.32 to 24.55)	0.68
	Cohort	4	56	21–91	99.44		
Quality of study	High	16	66	55–77	97.51	-2.78 (-14.58 to 9.01)	0.63
	Moderate	24	54	39–69	99.60		
	Low	10	75	53–96	99.48		
Sample size	< 50	17	62	46–77	96.43	0.07 (-17.55 to 17.71)	0.99
	> 50	33	662	51–73	99.62		
Year	< 2005	11	61	36–86	99.45	-6.75 (-14.10 to 0.59)	0.20
	2006–2010	13	71	53–90	99.61		
	2011–2015	13	71	56–86	97.15		
	> 2015	13	42	25–59	99.46		

*Footnote: Prevalence was provided per 100 people

## Discussion and conclusion

Our findings indicated that two-third of prisoners living with HIV are infected with HCV and one in ten are infected with HBV. Our results also indicated the hepatitis-HIV co-infection prevalence rates are highly variable across the globe.

In comparison with the prevalence of 7.0% for HBV and 2.4–5% for HCV among HIV positive individuals in the general population [2, 67, 68], we found a much higher prevalence of such co-infections among prisoners living with HIV. Several societal and environmental factors in prisons contribute to the higher transmission of HIV, HBV, and HCV infections among HIV-positive prisoners (these risk factors include population density, tattooing, drug injection, and sexual behaviors) [67, 69]. Also, we found a high prevalence of HIV/HCV co-infection (62%) among prisoners, in comparison to the prevalence of both HIV/HBV and HIV/HCV coinfections in health care workers (31%) [70]. In our study, we found that the prevalence of HCV/HIV co-infection in prosoners was significantly higher compared to high-risk groups in general population; including persons who inject drugs (PWID) (51%) [70] and 6.4% in MSM [71]. A possible justification for a higher rate of HCV than HBV in prisons could be attributed to the route of infection transmission [72]. For example, drug injection with sharing needles and syringes, and tattooing, which are substantially prevalent among prisoners, are strongly associated with transmission of HCV than HBV [72]. Also, using shared syringes and no vaccination for HCV among prisoners make them more prone to the acquisition of HCV than HBV [3]. More studies are needed to fully explain the possible reasons for the higher prevalence of HCV than HBV among prisoners with HIV.

Prisoners are considered as a high-risk group for HIV, HBV, HCV, and other sexually transmitted infections due to the more common risk factors such as drug injection with sharing needles and syringes, tattooing, and unsafe sex relationships. Prisons amplify adverse health conditions through overcrowding, poor infrastructure, and restricted access to healthcare services. Additionally, malnutrition, background infectious diseases, harsh social environment, and practices of some custodial officers toward inmates contribute to the deterioration of the physical and mental health of prisoners after incarceration [68, 73].

The current study found a higher pooled prevalence of HCV among HIV-positive prisoners who inject drugs (78%), a phenomenon mostly attributed to unsafe injection. A meta-analysis of 30 studies showed that prisoners who injected drugs are about 24-times more likely to be HCV infected than those with no history of drug injection [74]. Another study conducted on prisoners showed that HCV infection is the main risk factor associated with HIV infection with an odds ratio of 7.5 [43]. Researchers reported that the prevalence of HCV infection ranges from 22 to 40% among incarcerated populations, and that many prisoners acquire these infections before being incarcerated [75].

Regarding the geographical disparity in the rates of co-infections, the highest pooled prevalence of HCV/HIV co-infection was found in the South East Asia and East Mediterranean regions (83% for both regions) and the lower prevalence in America region (48%). Differences in the prevalence of HCV/HIV co-infection in prisons among different regions can be explained by some cultural, social, behavioral, and demographical differences. For example, more than 80% of the heroin production in the world is produced in Afghanistan, a country located in the East Mediterranean region [76] while injecting drug users (IDUs) is not common in the Caribbean [77]. Likewise, it is reported that, the proportions of in-prison drug injection are varying from 13% in Australia [78] to 61% in Mexico [79]. Results of a systematic review and meta-analysis revealed that behaviors like unsterile tattooing and body piercing, two risky behaviors that are common in prisons, are other sources of in-prison transmission of HIV, HBV, and HCV infections [80]. Also, more than 60% of inmates in Puerto Rican prisons get tattoos in prison with shared needles and unsterilized sharp objects [81].

According to our findings, the prevalence of HBV/HIV co-infection in prisoners has a wide variation from 3% in Eastern Mediterranean and South East Asia, to 27% in the Western Pacific region. The discrepancy between regions in the results of pooled prevalence of HBV/HIV co-infection is also reported among the general population [82] with the highest proportion in the West and Central Africa (12.4%) and the lowest proportion in Latin America and the Caribbean (5.1%). One study reported the prevalence of HBV/HIV as 15% in Africa [64]. However, there is limited data on the HBV/HIV co-infection in the African region, as only about 2% of prisoners are tested for HBV (before or during incarceration) in the region [83]. Also, only 14% of African prisoners have heard about hepatitis before being incarcerated, and HBV screening or vaccination is not a routine procedure in the prisons of this region [83].

Although limited data is available on the frequency and dynamics of risky sexual activities in detention centers and jails, it is suggested that, in the absence of access to condom and lubricants, sex for pleasure or in exchange for drug, money, personal protection, food, or other goods is common in prisons and this risky behavior is even more common among prisoners with HIV [3, 83].

The high prevalence of HBV and HCV among HIV-positive prisoners found in this study is a global alert for the necessity of providing specific and effective interventions in prisons. Guidelines for the implementation of comprehensive evidence-based interventions in prisons have been provided by both WHO and the UN Office on Drugs and Crime [84, 85]. Among some key interventions to prevent the transmission of blood-borne and sexually transmitted infections in prisons, screening and, if possible, vaccination and medical services (e.g. antiretroviral therapy (ART)), surveillance programs, health education, and consultation to prisoners or staff, free and easy access to condom, and lubricants for prisoners are essential. Services in prisons, including vaccination, health education, and screening at the entry of a prisoner, are crucial to diagnose, treat, and prevent the transmission of communicable diseases [85]. Although limmited evidence regarding the effectiveness of HCV and HBV screening among prisoners exist, a systematic review on routine screening for blood-borne viruses in prisons showed that routine opt-in and opt-out screening is both feasible and effective [86]. Although difficulties regarding the implementation of HIV and viral hepatitis screening in prison exist, new achievements in HCV treatment have led to the call for HCV case-finding and treatment for prisoners [87]. Also, for HBV, vaccination of HIV-positive individuals is recommended to reduce the risk of transmission of this infection. This conclusion is also supported by the results of a comprehensive review on this subject [88].

As mentioned before, despite the generally poor sanitary conditions and limited access to disposable syringes, drugs are always available in prisons. Knowing that people who inject drugs comprise about one-third to half of the prisoners and that needles and syringes are expensive to obtain in the prisons, use/reuse of unsafe injecting drug equipment is highly common and prisoners are often forced to share or make their injecting equipment [89]. As a result, there is mounting evidence that one of the most effective ways of preventing the spread of blood-borne infections in prisons is providing harm reduction services such as needle and syringe programmes (NSPs) and opioid substitution therapy (OST) [90]. Yet, the availability of these life-saving services remains extremely limited when compared to what is available in the community. For example, while 90 and 80 countries implement NSPs and OST in their community respectively, only seven and 43 implement these services in at least one of their prisons [91, 89].

Because the vast majority of prisoners will eventually return to the community, the prisoners’ health is intimately connected to public health. As a result, there is no doubt that to reach the WHO’s global targets on the control of HIV, tuberculosis (TB), and HCV, health and harm reduction services in prisons need to be significantly scaled up [89].

We believe, any comprehensive national and international strategy for the prevention, early diagnosis, and treatment of viral hepatitis must include jails and prisons. Immunization of those who are tested negative, treatment of those who are chronically infected, treatment of substance use in correctional facilities, and harm-reduction services can benefit any population by decreasing recidivism, infection transmission, and the costs associated with the treatment of chronic viral hepatitis in the population [92].

This study has some strengths and limitations to be noted. The important procedures such as searching studies, data extraction, and quality assessment were independently performed by two experts. However, there was limited data for some WHO regions in the subgroup analysis. Another limitation to the present study is publication bias, as some regions had more published studies than other regions. Finally, the difference in the HBV and HCV diagnosis methods may influence the estimated prevalence and observed heterogeneity.

In summary, our findings suggested a high prevalence of HBV and HCV coinfections among prisoners living with HIV particularly those with a history of drug injection that varied significantly across different regions. According to the results of the meta-regression, there was no reduction in the prevalence of the studied co-infections over the past decades, which call for better screening and treatment programs targeting prisoners living with HIV. We found that HCV/HIV coinfection was more prevalent than HBV/HIV coinfection in the prisons especially if they were IDUs. The Key action is urgently needed to scale up evidence-based interventions to prevent HIV, HBV, and HCV in prisoners by a broad range of strategies. Cooperation and collaboration will be needed to take place between the governments, justice departments, the prisons’ staff, health-care workers, academics, and NGOs. The findings of the current study are highly valuable for policymakers to design and implement interventional programs among high-risk groups especially prisoners.

## Supplementary Information

**Additional file 1: Figure 1.** Map of Hepatitis B co-infection among prisoners living with HIV by country within different WHO regions (pooled prevalence and 95% CI are shown for each country). **Figure 2.** Funnel plot for publication bias of the included studies on HBV/HIV co-infection. **Figure 3.** Egger test for prevalence of HBV among HIV patients. **Figure 4.** Meta-regression of HBV infection prevalence over time among prisoners living with HIV. **Figure 5.** Funnel plot for publication bias of the included studies on HCV/HIV co-infection. **Figure 6.** Egger test for prevalence of HCV among HIV patients. **Figure 7.** Meta-regression of the association between the prevalence of HCV/HIV coinfections during 1990–2019 in HIV-positive prisoners and year of study.

## Data Availability

The data are presented in the manuscript.

## Abbreviations

*AIDS*: Acquired immunodeficiency syndrome
*ART*: Antiretroviral therapy
*CI*: Confidence interval
*HIV*: Human immune deficiency virus
*HCV*: Hepatitis C virus
*HBV*: Hepatitis B virus
*IDUs*: Injecting drug users
*NSPs*: Needle and syringe programmes
*OST*: Opioid substitution therapy
*PWID*: Persons who inject drugs
*PRISMA*: Preferred reporting items for systematic reviews and meta-analyses
*SE*: Standard error
*TB*: Tuberculosis
