# Supplementary material for: The global and regional prevalence of hepatitis C and B co-infections among prisoners living with HIV: a systematic review and meta-analysis
Source: Infect Dis Poverty. 2021 Jul 1;10:93. doi: 10.1186/s40249-021-00876-7 (PMC8252262; doi:10.1186/s40249-021-00876-7)
Supplement: Supplementary file 1 — Additional file 1: Figure 1. Map of Hepatitis B co-infection among prisoners living with HIV by country within different WHO regions (pooled prevalence and 95% CI are shown for each country). Figure 2. Funnel plot for publication bias of the included studies on HBV/HIV co-infection. Figure 3. Egger test for prevalence of HBV among HIV patients. Figure 4. Meta-regression of HBV infection prevalence over time among prisoners living with HIV. Figure 5. Funnel plot for publication bias of the included studies on HCV/HIV co-infection. Figure 6. Egger test for prevalence of HCV among HIV patients. Figure 7. Meta-regression of the association between the prevalence of HCV/HIV coinfections during 1990–2019 in HIV-positive prisoners and year of study. [file 40249_2021_876_MOESM1_ESM.docx]

**Additional files**

**The Global and Regional Prevalence of Hepatitis C and B Co-infections among Prisoners Living with HIV: A Systematic Review and Meta-Analysis**

Hasan Ahmadi Gharaei, Ali Mirzazadeh, Golnaz Sharifnia, Marziyeh Rohani-Rasaf, Dariush Bastam, Jamileh Rahimi, Mostafa Kouhestani, Shahab Rezaian, Mohammad Fararouei, Mostafa Dianatinasab^*^

Details of an eight-item checklist for critically appraisal studies of prevalence/incidence of a health problem is provided as follow:

This tool defines 8 criteria namely: (1) whether a random sample or whole population was used, (2) if an unbiased sampling frame was used, (3) adequacy of the sample size, (4) the use of standard measures, (5) whether outcome measurements were made by unbiased assessors, (6) adequacy of the response rate, (7) confidence intervals (CIs) and subgroup analyses, and (8) whether the study subjects were described. Each item was scored as 1 if a study met the criterion and zero otherwise, and the scores were summed up.

_______________________________________________________________________________


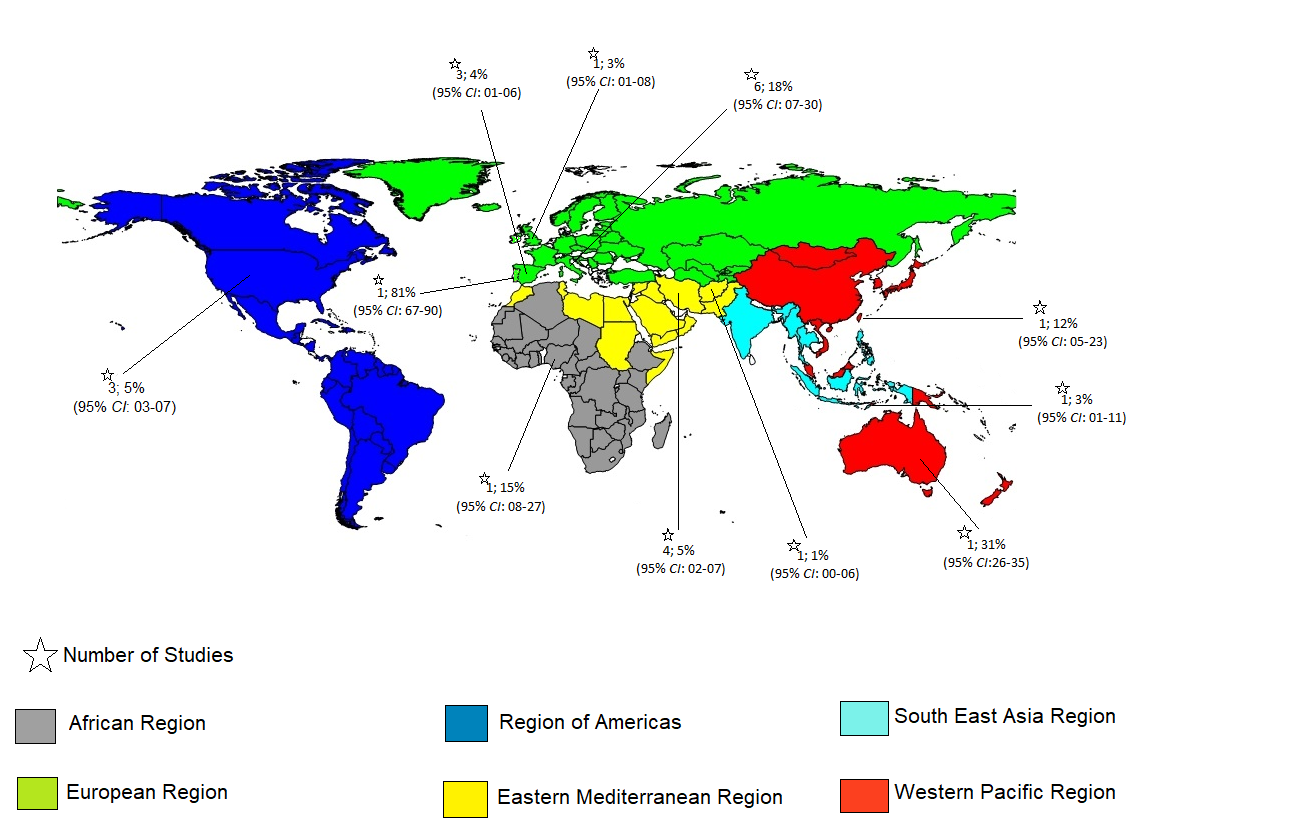


*Footnote: Prevalence was provided per 100 people. "*" is shown number of studies.*

**Additional file-Figure S1**. Map of Hepatitis B co-infection among prisoners living with HIV by country within different WHO regions (pooled prevalence and 95% CI are shown for each country).

******

**Additional file-Figure S2.** Funnel plot for publication bias of the included studies on HBV/HIV co-infection.

**Additional file-Figure S3.** Egger test for prevalence of HBV among HIV patients.

******

**Additional file-Figure S4.** Meta-regression of HBV infection prevalence over time among prisoners living with HIV.

**Additional file-Figure S5.** Funnel plot for publication bias of the included studies on HCV/HIV co-infection.

**Additional file-Figure S6.** Egger test for prevalence of HCV among HIV patients.

******

**Additional file-Figure S7.** Meta-regression of the association between the prevalence of HCV/HIV coinfections during 1990-2019 in HIV-positive prisoners and year of study.
